# Cardiac Rehabilitation and Cardiopulmonary Fitness in Children and Young Adults With Congenital Heart Diseases: A Critically Appraised Topic

**DOI:** 10.7759/cureus.31483

**Published:** 2022-11-14

**Authors:** Ayoola Awosika, Angela R Hillman, Richard M Millis, Mayowa J Adeniyi

**Affiliations:** 1 College of Health Sciences and Professions, Ohio University, Athens, USA; 2 Department of Pathophysiology, American University of Antigua, St. Johns, ATG; 3 Department of Physiology, Edo University, Iyamho, NGA

**Keywords:** congenital heart disease, aerobic capacity, physical fitness, exercise training, cardiac rehabilitation

## Abstract

Public health guidelines and a myriad of studies have proven that exercise is beneficial in the alleviation of various cardio-metabolic diseases. Congenital heart disease (ConHD) is one of the most frequently occurring congenital structural malfunctions in the pediatric population, affecting nine of every 1,000 live births. Only a few studies have established the impact of a structured exercise program on cardiopulmonary fitness in diverse groups of patients with ConHD. It is also alarming to know that a substantial number of these patients and their caregivers often remain very wary of exercise. Anxiety about exercise may increase the risk of developing morbid obesity and other long-term health complications of ConHD. The present review of a critically appraised topic is undertaken to answer the question, “Does structured exercise intervention (cardiac rehabilitation) improve cardiorespiratory fitness in children and young adults with ConHD?” Exercise science and the medical literature were searched for studies that engaged the use of aerobic exercise in patients with different ConHD diagnoses. The search yielded four studies after screening with the inclusion and exclusion criteria, which were further narrowed to three studies after a full-text review. These studies yielded results showing significant increments in peak exercise workload, duration, power output, peak oxygen uptake, or improved tissue oxygenation and muscle strength after an exercise training intervention. It is noteworthy that a group identified as “cyanotic palliated” exhibited the most significant impairment both at baseline and after the exercise intervention. This review provides level 1b medical evidence that a structured exercise program may improve cardiopulmonary fitness in patients with ConHD, which is likely to be beneficial to their overall physical, motor, and psychosocial development. The results of this review may be useful for alleviating the anxiety of patients and their caregivers about participation in structured exercise programs. This review should also motivate future research investigations to develop clinical guidelines for the management of patients with ConHD by adding exercise prescriptions to their daily therapeutic regimens.

## Introduction and background

**Figure 1:**
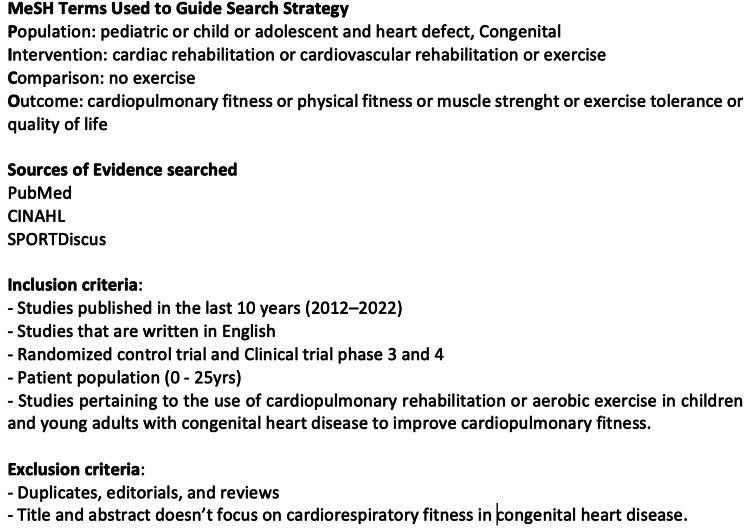
Search strategy and criteria. A detailed description of PubMed, CINAHL, and SPORTDiscus scientific literature database searches employing patient/population, intervention, comparison, and outcome (PICO) criteria for evidence-based medicine investigations. MeSH = Medical subject headings used to index PubMed articles.

Congenital heart disease (ConHD) is a group of structural and functional abnormalities of the heart or great vessels that are present at birth [1]. These malformations result in abnormal communication between the heart chambers or blood vessels which can eventually lead to a reduction in oxygenation, poor circulation of nutrients, and/or general hemodynamic instability. The incidence of ConHD is greater in premature than in full-term newborns, approaching about 1% of the pediatric population [2]. With the help of surgical and medical treatment, about 80% of children with ConHD can survive into adulthood [3]. Public health guidelines generally recommend that patients with ConHD should exercise as tolerated [4], to help reduce long-term complications associated with their medical condition and to improve health-related quality of life and overall physical fitness [5]. However, there is often a reluctance to exercise in patients with ConHD due to the perceived fear of exercise exertion causing more harm to their hearts [6]. It is well established that patients with ischemic heart disease participating in cardiac rehabilitation programs experience significant improvement in their conditions [7]. Improvement in cardiopulmonary fitness is reported as the main therapeutic outcome of cardiac rehabilitation that is, in general, responsible for the reduction in morbidity and mortality for cardiac patients [8-11]. Based on a Cochrane review of physical activity interventions, it is discovered that aerobic exercise training results in small to modest improvements in prognosis for patients with ConHD; whereas, the long-term effects may yield little evidence for a change in their prognosis [12]. The aim of this review is to search, review and critically appraise the medical and other scientific literature addressing the impact of structured exercise intervention (cardiac rehabilitation), in order to determine whether there is any appreciable improvement in cardiopulmonary fitness of patients living with ConHD. The search strategy and criteria are presented in Figure 1.

Search results

**Figure 2:**
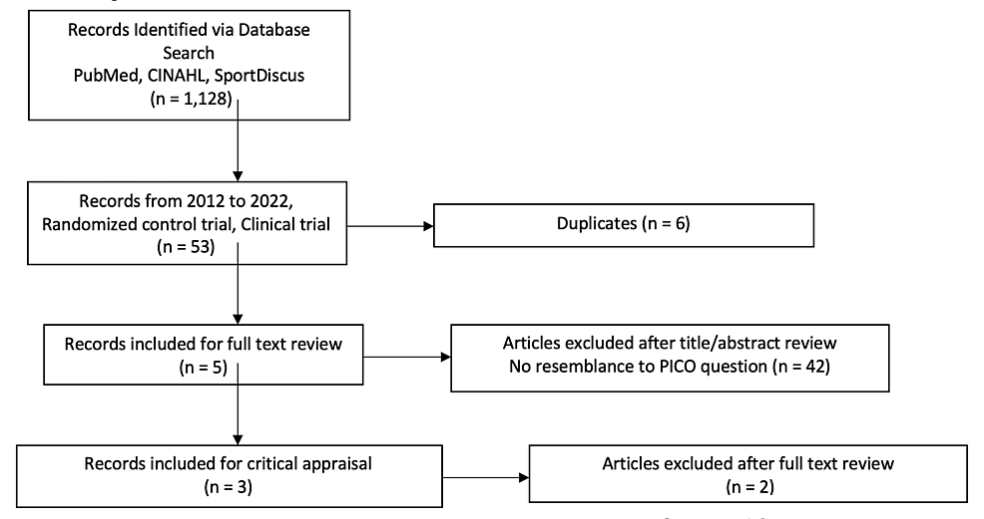
Search results. PubMed = National Library of Medicine (USA) database of medial and biomedical research literature. CINAHL = Cumulative Index to Nursing and Allied Health Literature. SPORTDiscus = Bibliographic database of exercise and sports medicine research. PICO = patient/population, intervention, comparison, and outcome criteria employed for ensuring the relevance of the information for evidence-based investigations.

Exercise science and the medical literature were searched for studies that investigated the effects of cardiopulmonary rehabilitation on cardiopulmonary fitness in children and young adults with ConHD. Figure 2 summarizes these search results. 1,072 articles were eliminated based on the inclusion or exclusion criteria. An additional 42 articles did not address the research question relevant to the patient/population, intervention, comparison, and/or outcome associated with the PICO search strategy as described in Figure 1.

Table 1 summarizes the findings of four articles found to be highly relevant to this appraisal based on the inclusion criteria [13-16].

**Table 1:** Evidence-based assessment of the most relevant studies. ConHD=congenital heart disease; TOF=tetralogy of Fallot; EST=exercise stress test; MVPA=moderate to vigorous physical activity; MVC=maximum voluntary contraction; VO2=oxygen consumption; MET=metabolic equivalent of task; MO2=muscle oxygen consumption; NIRS=near infrared spectroscopy; DmO2=pre-post-training difference in muscle oxygen consumption; Tlim=endurance time; PEDro=Physiotherapy Evidence Database of randomized clinical trials.

Citation	Callaghan et al. 2021 [13]	Duppen et al. 2015a [14]	Duppen et al. 2015b [15]	Moalla et al. 2012 [16]
Study design	Prospective RCT	Multicenter prospective RCT	Multicenter prospective RCT	Prospective RCT
Population	163 patients with ConHD Control group n=81 Intervention group n=82	93 patients with ToF or Fontan circ. Control group n=37 Intervention group n=56	48 patients with ToF or Fontan circ. Control group n=20 Intervention group n=28	18 patients with ConHD + surgical correction Control group n=8 Intervention group n=10
Inclusion/exclusion criteria	Inclusion: age 5-10 y, ConHD Exclusion: Cardiac diagnosis where it would be considered unsafe to participate in even moderate physical activity Major learning difficulty or serious comorbidity	Inclusion: age 10-25 y, ToF corrected by transatrial-transpulmonary approach, done before 3.5 y age Fontan circulation completed before 6 y age Exclusion: Right ventricular outflow obstruction peak >60 mmHg	Inclusion: age 10-25, ToF corrected by transatrial-transpulmonary approach Exclusion: Right ventricular outflow tract obstruction peak >60 mmHg. Diagnosis of movement, mental, or other disorders that impair cognition	Inclusion: age 12-15 y, Complex ConHD with ejection fraction < 40% Surgically corrected pulmonary valve atresia (Fontan), ToF with right ventricular outflow construction, atrial septal defect and transposed great vessel Exclusion: Implanted pacemaker, diagnosis of movement, mental, or other disorders
Intervention investigated	Control & treatment groups had baseline biophysical assessment and EST with daily activity monitoring using accelerometer Treatment group interventions: 1. Individualized exercise advice plan MVPA averaged 45 min/d 2. Educational program (motivational interviewing) 3. Information package Reassessment of both groups after 4-month intervention	Controls continue normal lifestyle Treatment group had 12 weeks of a standardized aerobic exercise activity	Controls continue normal lifestyle Treatment group had 12 weeks of standardized aerobic exercise activity. Heart rate submaximal range set at 60 – 70% heart rate reserve .	Treatment group had 3 training sessions/week for 12 weeks on a stationary cycle ergometer Warm-up exercise 10 min unloaded cycling, 45 min loaded cycling, 5 min cool-down for active recovery (unloaded cycling)
Outcome measures	Peak exercise capacity & daily activity	Peak VO2 & daily physical activity level measured in MET	Peak VO2 and workload Echocardiographic measures of global & regional cardiac function	MVC measure of muscle strength, DMO2 by NIRS
Results	Cyanotic palliated group had shorter exercise duration (p=0.001) and lower power output (p=0.002) at peak exercise Time spent in MVPA/d less in the cyanotic group compared to acyanotic no intervention p<0.001 and to acyanotic repaired groups p<0.001 After 4 months of intervention: Significant increase in exercise duration in intervention group compared to control group p<0.001, effect size 43s, 11% increase over baseline Also, intervention group had significant increase in max. power output p<0.001	Exercise group: 5% increase in peak VO2 1.7 ± 4.2 vs 0.9 ± 5.2 mL/kg/min, p=.01 Significant increase in workload 6.9 ± 11.8 vs 0.8 ± 13.9 W; p <.05 no significant change in percentage of time mvpa at baseline	Peak workload: Exercise group- 170 ± 53 W vs controls 170 ± 38, not significant Significant increase in peak workload seen in exercise group after intervention delta pre vs post 8.4 ± 11.4 W vs 0.1 ± 15.9 W controls p=0.048 Significant increase in peak VO2 for exercise group delta 2.9 ± 4.0 mL/kg/min within-subject analysis, p=0.002 vs controls peak VO2 did not change significantly delta 0.7 ± 5.1 mL/kg/min within-subject analysis p=0.54	Exercise group: significant differences between pre- and post-training, for both MVC (101.6 ± 14.0 vs 120.2 ± 19.4 N·m, p< 0.01 and Tlim 66.2 ± 22.6 vs 86.0 ± 23.0 s, p < 0.01, not significant for controls Training group had significant increase in both DMO2 and mean rate of decrease in VO2, indicating improved O2 usage p< 0.001 Significant correlations in exercise group between delta scores (post - pre) of both DMO2 and Tlim (r = 0.90, p< 0.001) and DMO2 and MVC r=0.95, p< 0.001
Level of evidence	1b	1b	1b	1b
Quality assessment score (PEDro)	8	9	8	8
Contribution to answering CAT question	Structured intervention program (cardiac rehabilitation) has significant impact in improving peak exercise capacity in children with ConHD	Patients with ToF show improved physical performance during aerobic exercise training No improvement in Fontan patients No significant changes in daily physical activity in both groups	Aerobic exercise training does not impair regional systolic or diastolic ventricular performance after ToF correction	Individualized training program of cardiac rehabilitation is beneficial to increasing peripheral muscle use of oxygen during exercise and recovery, increasing muscular strength and endurance in patients with ConHD

The articles summarized in Table 1 were critically assessed based on the physiotherapeutic evidence database of randomized control trials (PEDro), widely recognized as a valid measure of the methodological quality of RCTs [17].

## Review

Implications for practice, education, and future research

The four relevant studies identified by this review involved the use of aerobic exercise activity in patients who had ConHD and no adverse medical events occurred during the studies. The Callaghan et al. study, the largest randomized control trial, investigated exercise might impact children afflicted with ConHD [13]. Overall, after a baseline biophysical assessment, participants in the intervention group showed an 11% increase in exercise duration, coupled with an increase in maximum power output and daily activity. This study divided patients into intervention groups based on their spectrum/severity of ConHD. An acyanotic group included patients diagnosed with atrial septal defect (ASD), ventricular septal defect (VSD), pulmonary stenosis, and aortic coarctation while a cyanotic group was comprised of conditions including transposition of great vessels (TGA), tetralogy of Fallot (TOF), total anomalous pulmonary venous return (TAPVC), persistent truncus arteriosus and pulmonary atresia. A cyanotic palliated group was comprised of patients with right ventricle hypoplasia, left heart hypoplasia, and tricuspid atresia. Of the three intervention groups, the cyanotic palliated group demonstrated the most significant cardiorespiratory impairment during exercise both at baseline and after the intervention. They had lower power output, shorter exercise duration at peak exercise (EST duration 4.54±1.88 min), lower average time spent in mild to vigorous physical activity (MVPA) each day (34.48±23.67 min); and their average daily step count (8,665 steps) was below all the groups. This study involved clinical observations of children with ConHD, revealing increasing concerns about a decline in daily physical activities which could translate into a higher prevalence of obesity when compared to their contemporaries [18]. There is also an increased risk for poor long-term outcomes that could adversely affect their physical, motor, and psychosocial development [19]. Another distinctive approach of the Callaghan et al.'s study was the deployment of motivational interviewing techniques when delivering an individualized exercise advice plan to the patients in the intervention group [13]. Such initiatives are encouraged by the American Heart Association because it is thought to promote behavioral changes and adherence to structured exercise programs which can encourage better future habits, especially within this patient population who are known to have a low motivation for exercising [20]. 

Duppen et al. assessed the impact of a 12-week regimen of aerobic exercise training on the daily activities of patients with corrected TOF and more complex ConHD, such as in the Fontan circulation [14]. A total of 93 subjects were involved in the randomized control trial wherein an intervention group was subjected to a 1h daily exercise session, consisting of 10 min of warm-up, 40 min of aerodynamic cardiovascular training, and 10 min of cool-down. The findings revealed that peak oxygen consumption (VO2) increased by 5% in the intervention group (p=0.011) compared to the controls. Other parameters such as workload, ventilation (peak VE), and peak oxygen pulse also showed significant increments when compared to the controls. One of the more interesting aspects of this study is that despite the fact that the TOF exercise group had significant increases in various cardiorespiratory outcome parameters, the Fontan intervention group did not demonstrate a significant increment in cardiorespiratory fitness. This finding was corroborated by the lack of an increase in stroke volume during a dobutamine stress assessment; thereby, suggesting that a Fontan circulation predisposes patients to preload impairment and a subsequent limitation in exercise capacity.

An additional report by Duppen et al. is relevant. This study focused on the impact of a three-month regimen of aerobic dynamic exercise on ventricular performance in patients with TOF [15]. Previous studies have shown that any systolic and diastolic ventricular performance changes could be good physiologic markers for monitoring any decline in cardiorespiratory function in these ConHD patients [21]. The Duppen et al.'s study involved aerobic activity three times per week, consisting of 1h of exercise training at a submaximal level with 10-min warm-up intervals, 40-min of cycling exercise, and another 10-min period of cool-down exercise [15]. Peak workload and peak VO2 were used as surrogate markers to gauge cardiopulmonary fitness. The intervention group had a significant increase in peak workload when compared to baseline (delta exercise group 8.4±11.4 Watts Vs change observed in the control group (-0.1±15.9 Watts; p=0.048). Peak oxygen consumption also increased significantly in the TOF exercise group (delta 2.9±4.0 mL/kg per min; within-subject analysis, p=0.002) with a minimal difference in peak VO2 in the control group. An interesting aspect of this study involved children with highly abnormal loading conditions of the heart. This study revealed no evidence of training-related adverse effects on systolic and diastolic cardiac performance. The result of this study bolsters the current public health guidelines and body of evidence that supports the participation of children with ConHD in exercise activities [22], as well as encouraging the physicians who are treating these children to recommend structured exercise programs for this patient population.

Moalla et al. investigated the impact of an individualized training program during cardiac rehabilitation on peripheral muscular performance and oxygenation during exercise and recovery [16]. This study engaged a broad spectrum of children with surgically corrected complex ConHD. Both exercise and control groups were well-matched in age, body mass, and height. The exercise intensity regimen was established by using cardiopulmonary exercise testing (CPET) to individualize exercise tolerance and capacity. An isokinetic dynamometer was employed to determine the muscular strength during knee extensors exercise sessions, reported as a maximal voluntary contraction (MVC) and a time-limited measure of muscle endurance (Tlim) at 50% MVC before and after the exercise training sessions. Muscle oxygenation was measured by near-infrared spectroscopy (NIRS) at 50% MVC. time to exhaustion, Tlim, and recovery. Each training session consisted of aerobic cycling involving a 10-min warm-up interval (unloaded cycling), a 45-min loaded cycling, and a 5-min cool-down period for active recovery. This study revealed a significant improvement in the following parameters after the training intervention vs. the control group): an increase in MVC (101.6 ± 14.0 vs. 120.2 ± 19.4 N·m, p < 0.01), an increase in Tlim (66.2 ± 22.6 vs. 86.0 ± 23.0 s, p < 0.01), an increase in muscle oxygenation (0.20 ± 0.13 vs. 0.15 ± 0.07 a.u., p < 0.01), and a faster rate of decrement in muscle oxygenation (1.22 ± 0.45 vs. 1.71 ± 0.78%·s-1, p < 0.001), all of which are indicative of an improvement in peripheral muscle oxygen utilization during exercise and recovery.

The significance of these findings of the Moalla et al.'s study is underscored by reports that fatigue and exercise intolerance are common complications in patients with ConHD due to a limited supply of energy to peripheral muscle tissues [23]. This energy supply limitation is likely to result from impaired blood flow, thereby leading to insufficient oxygenation during exercise. The findings of the Moalla et al.'s study also suggest an interplay of cardiorespiratory and peripheral muscular factors. Previous studies have also shown that peripheral muscle functions play a substantial role and are strong determinants of reduced exercise capacity [24]. The findings of Moalla et al. suggest that individualized aerobic training programs may go a long way in improving peripheral muscle oxygenation; and therefore, exercise tolerance in patients with ConHD, which might ultimately translate to improvements in functional capacity, life expectancy, and other benefits related to the quality of life. The pathophysiologic basis for such improvements could be attributed to hemodynamic factors such as increased cardiac output during exercise, improved autonomic function, decreased myocardial oxygen demand, and/or delayed ventilatory threshold onset. The peripheral contribution may also stem from optimized endothelial function, decreased vessel resistance, improved blood flow redistribution, increased capillary and mitochondria density, improved phosphocreatine energy supply systems during exercise and recovery, as well as increased efficiency in arteriovenous oxygen extraction.

Translating these findings into safe clinical practice

This review suggests that exercise is recommended and safe for all categories of patients with ConHD. However, patients with surgically corrected, palliated the Fontan operation or uncorrected cyanotic heart defects need to be closely supervised because of the obvious risk of sudden oxygen desaturation [25]. It is, therefore, imperative to select an appropriate exercise protocol that does not exacerbate existing or create additional, hemodynamic instability. The rate of exercise progression should be based on a patient’s symptoms, signs of overexertion, rate of perceived exertion (RPE) between 11 and 14 on a scale of 6 to 20, and clinical judgment. In practice, this goal can usually be achieved by gradually increasing the patient’s workload, as tolerated. An exercise prescription or protocol that includes more aerobic, and less resistance, training is recommended because aerobic exercise limits the elevations of systemic vascular resistance and end-diastolic pressure which have the potential to limit exercise tolerance by decreasing stroke volume, as is more often associated with resistance training [11]. Previous studies have also shown that administering supplemental oxygen enhances exercise performance in cyanotic ConHD [26]. Oxygen supplementation permits a longer duration of activity initially, with the goal of gradual withdrawal, as tolerated. As a gold standard, employing CPET to safely assess the functional capacity of each ConHD patient prior to initiating their cardiac rehabilitation program is shown to be a reliable predictor of exercise tolerance and prognostic tool [27].

## Conclusions

The present review of the exercise science and medical literature was undertaken to critically appraise the topic of whether participation in a structured exercise program may provide more benefits than risks to patients living with ConHD. Based on two relevant studies, involving 256 patients, suggest that participation in a structured exercise program of cardiac rehabilitation may have a positive impact on improving cardiopulmonary fitness in patients with ConHD, without adding substantial risk for adverse cardiac events or other health risks. Children with complex ConHD are at increased risk of being limited to lower-intensity activity compared to their peers. Structured exercise for cardiac rehabilitation is considered a medical treatment and, as with any medical treatment, early identification and intervention are recommended to help prevent deconditioning. This review may offer help in addressing the concerns of caregivers and patients about the perceived fear of exercise exertion worsening their heart condition. The research cited in this review suggests that an increase in exercise duration, workload, power output, and peak oxygen uptake may be beneficial when compared to patients who never exercised. Having established the efficacy of aerobic activity in this patient population, future studies should be designed to determine the frequency and duration of activity that patients should engage in to achieve optimum cardiopulmonary fitness safely. This critically appraised topic should be revisited frequently to assess future research evidence that could be useful in the management of ConHD.
